# The relationship between parental involvement matching and adolescents’ internal locus of control: a response surface analysis

**DOI:** 10.3389/fpsyg.2026.1835772

**Published:** 2026-06-12

**Authors:** Hongbo Cui, Yuxing Wang, Yue Tian, Zhen Zhou, Yanming Lv, Xiaoshan Lin

**Affiliations:** 1 School of Education, Guangzhou University, Guangzhou, Guangdong, China; 2 College of Political Science and Public Administration, Henan Normal University, Xinxiang, China; 3 School of Computer Science and Network Engineering, Guangzhou University, Guangzhou, Guangdong, China; 4 School of Artificial Intelligence, Guangzhou University, Guangzhou, Guangdong, China

**Keywords:** adolescents, internal locus of control, matching, parental involvement, response surface analysis

## Abstract

This study analyzes the effects of congruence and incongruence in paternal and maternal involvement on adolescents’ internal locus of control. A sample of 680 adolescents was assessed using the Parental Involvement Behavior Questionnaire and the Internal Locus of Control Scale, and response surface analyses were conducted. Results revealed that: (1) Under conditions of parental involvement congruence, adolescents exhibited significantly higher levels of internal locus of control in low paternal–low maternal involvement dyads compared to high paternal–high maternal involvement dyads. (2) Under conditions of parental involvement incongruence, adolescents demonstrated higher internal locus of control in high paternal–low maternal involvement dyads versus low paternal–high maternal involvement dyads. The findings indicate that dual-high parental involvement is associated with lower internal locus of control compared to low-low involvement, while higher paternal involvement in high paternal–low maternal configurations correlates with higher internal locus of control.

## Introduction

Locus of control, a cornerstone concept in social learning theory, refers to an individual’s generalized expectancy regarding whether life outcomes are contingent upon their own actions or external forces, reflecting a stable attributional style that permeates cognitive, emotional, and behavioral domains (Rotter, 1966; Ajzen, 2002). Rooted in Heider’s attribution theory, which emphasizes how individuals infer causality in social interactions, and Weiner’s causal attribution framework, which links attributions to motivation and emotional responses, Rotter (1966) formalized the construct of locus of control as a continuum spanning internal and external orientations. Internal Locus of Control (ILOC)—defined as the tendency to attribute outcomes to one’s own efforts, abilities, or choices—emerges as a pivotal psychological resource, particularly during adolescence, a developmental stage marked by rapid identity formation, heightened autonomy needs, and increasing responsibility for life decisions (Güzel et al., 2024).

Adolescents with a strong internal locus of control demonstrate distinct adaptive advantages: they are more likely to attribute academic success or failure to controllable factors (e.g., effort, strategy) rather than external forces (e.g., luck, task difficulty), thereby engaging in proactive learning behaviors such as seeking feedback or adjusting study methods to improve performance (Chukwuorji et al., 2018; Cui et al., 2023). Beyond academic contexts, internal locus of control correlates with greater emotional resilience, as individuals with internal control beliefs are better equipped to cope with stress by focusing on actionable solutions (Naghsh et al., 2023). They also exhibit lower engagement in risky behaviors (e.g., substance use, delinquent acts), as they perceive a stronger link between their choices and negative consequences (Georgiou et al., 2017). Given these far-reaching impacts, understanding the factors that shape internal locus of control during adolescence is critical for promoting healthy development and mitigating developmental risks.

Consistent with Bronfenbrenner’s (1986) ecological systems theory, the family constitutes the most proximal and influential microsystem in adolescents’ lives, exerting profound effects on psychological development through daily interactions, role modeling, and emotional dynamics. Notably, Yang and Wang (2008) found that environmental factors—including family experiences—account for a larger proportion of variance in locus of control development than genetic predispositions, underscoring the importance of investigating family contextual influences. Within the family system, parental involvement emerges as a key variable, defined as the intentional cognitive, emotional, and behavioral investments by fathers and mothers to support their children’s growth across physical, cognitive, and socioemotional domains (Lamb, 2010; Boonk et al., 2018; Frank et al., 2010).

In contemporary societies, escalating educational competition and parental anxiety have transformed the landscape of parental involvement, giving rise to phenomena such as “rat race parenting” (Rienecke, 2020) and “involution in education” (Zhou and Cui, 2025) in East Asian contexts like China. This trend is characterized by excessive time, energy, and financial investments in children’s academic and extracurricular activities, often driven by a desire to secure competitive advantages. While numerous studies have highlighted the benefits of parental involvement, including strengthened parent–child bonding (Timperley et al., 2022), enhanced children’s self-efficacy (Williams et al., 2017), and positive associations with internal locus of control (Ahlin and Lobo Antunes, 2015; Yang et al., 2024). Paradoxically, a growing body of studies have also revealed a “diminishing returns” effect in some families, that is, beyond a certain threshold, increased parental involvement fails to yield proportional benefits and even may be linked to reduced development (Leung and Shek, 2014; Luthar et al., 2020). This paradox is particularly salient during adolescence, a period of recalibrated parent–child dynamics. As adolescents strive for autonomy and independence, the relationship shifts from a hierarchical, authority-based structure to one of mutual negotiation (Steinberg and Silk, 2002; McGue et al., 2005). In this context, parental involvement that is perceived as intrusive or controlling—rather than supportive—may correlate with a weaker adolescents’ sense of agency. Cultural factors are associated with greater this tension in Chinese families: traditional norms emphasize restraint in emotional expression, leading Chinese parents to exhibit lower levels of overt warmth compared to Western parents (Rothenberg et al., 2020). Consequently, high parental involvement may be interpreted by adolescents not as care, but as surveillance or pressure, creating a perceptual gap between parental intentions and adolescent experiences (Leung and Shek, 2014; Kapetanovic and Boson, 2022). Such misalignment can erode self-efficacy (Youn et al., 2023; Zhou and Cui, 2025) and, by extension, impede the development of internal locus of control.

Family systems theory further illuminates the complexity of parental involvement by framing the family as an interconnected network where the behaviors of one subsystem (e.g., fathers) influence and are influenced by others (e.g., mothers, children; Cox and Paley, 2003). This perspective underscores that paternal and maternal involvement should not be studied in isolation but as a dyadic system, as their combined patterns—whether congruent (e.g., both high or both low) or incongruent (e.g., father high-mother low or vice versa)—may exert unique effects on adolescent outcomes. Despite this theoretical imperative, existing research has predominantly focused on “overall” parental involvement, neglecting potential differences between fathers and mothers (Ahlin and Lobo Antunes, 2015; Yang et al., 2024). This oversight is particularly striking in Chinese cultural contexts, where traditional gender roles structure distinct parental responsibilities: fathers are often cast as “instrumental providers” (e.g., academic guidance, rule-setting), while mothers are viewed as “emotional caregivers” (e.g., daily care, affection; Leung and Shek, 2014; Liu et al., 2023; Zheng et al., 2025). These role expectations shape both parental behavior and adolescent perceptions. For instance, culturally normalized high maternal involvement may be taken for granted or even perceived as intrusive, whereas paternal involvement—historically less common due to the “breadwinner” norm (Li, 2020)—may carry greater psychological weight when it occurs, as adolescents interpret it as intentional and voluntary support (Zhou and Cui, 2025). Such dynamics suggest that paternal involvement could play a uniquely critical role in fostering adolescents’ internal locus of control, though this hypothesis remains untested.

Methodological limitations further constrain current understanding. Most studies examining parental involvement congruence rely on difference scores (e.g., paternal minus maternal involvement) or cluster analyses, which obscure nuanced patterns. Difference scores, for example, collapse directional and magnitude differences into a single value, making it impossible to distinguish between “father high-mother low” and “father low-mother high” dyads (Edwards, 2002; Edwards and Cable, 2009). Response Surface Analysis (RSA), by contrast, models the joint effects of two predictors (e.g., paternal and maternal involvement) as a three-dimensional surface, enabling precise quantification of how congruence (*X* = *Y*) and directional incongruence (*X* > *Y* vs. *X* < *Y*) relate to outcomes (Shanock et al., 2010). This method has been successfully applied to unpack parent–child dynamics, such as perceptual congruence in warmth and control (de Haan et al., 2018; Janssen et al., 2021), but has rarely been used to explore parental involvement patterns and their links to internal locus of control—especially in non-Western contexts.

In summary, three critical gaps persist in the literature: (1) the lack of attention to dyadic patterns of paternal and maternal involvement, particularly in Chinese cultural contexts with distinct gender role norms; (2) insufficient understanding of how congruent versus incongruent involvement shapes adolescent internal locus of control; and (3) overreliance on methodologically limited approaches (e.g., difference scores) that fail to capture complex dynamics. The present study addresses these gaps by employing RSA to investigate how paternal and maternal involvement patterns—both congruent and incongruent—influence adolescents’ internal locus of control. By visualizing these relationships through three-dimensional surfaces, we aim to clarify whether certain involvement configurations (e.g., high-high, low-low, high-low, low-high) are more conducive to internal locus of control development, thereby advancing theoretical understanding and providing culturally relevant insights for parenting practices. The theoretical contributions of this study are threefold: first, it advances the field by adopting a dyadic family system perspective to examine parental involvement congruence and incongruence, moving beyond the uniparental framework adopted in most prior studies; second, it employs Response Surface Analysis to clarify the directional and nonlinear effects of paternal and maternal involvement, overcoming the methodological limitations of traditional difference scores; third, it provides culturally specific evidence from Chinese adolescents, enriching the cross-cultural applicability of locus of control and parenting theories.

## Materials and methods

### Participants

We selected three public junior high schools in urban Guangdong Province as our sampling frame, adopting cluster sampling by class to cover Grades 7 to 9. These schools are regular compulsory education institutions with diverse student sources, representing the general characteristics of urban adolescent parenting in Guangdong. After obtaining consent from school authorities and students themselves, we administered the survey at the class level. The study strictly adhered to ethical guidelines throughout the process. Following written informed consent from participants and their guardians, homeroom teachers facilitated classroom-based group administration, with immediate retrieval of completed questionnaires.

A total of 750 questionnaires were distributed to the selected participants. After collecting the completed questionnaires, we carefully checked for the quality of responses. Through rigorous screening: (1) Questionnaires exhibiting response patterning or random responding were excluded; (2) Participants with non-parental primary caregivers were removed. No missing data existed in the valid sample, and the data quality was strictly guaranteed. The final valid sample comprised 680 participants (effective response rate: 90.7%), including 353 girls and 327 boys. Grade distribution: Grade 7 (*n* = 246), Grade 8 (*n* = 225), Grade 9 (*n* = 209). Mean age was 13.46 years (SD = 1.31).

### Measures

#### Parental involvement behavior questionnaire

Parental involvement was assessed using the Parental Involvement Behavior Questionnaire developed by Wu et al. (2018). This scale included four subscales: living care, discipline instruction, cognitive support and emotional interaction. Both father- and mother-targeted versions contain 22 identical items rated on a 5-point Likert scale (from 1 = never to 5 = always). The examples of items are: “Father/mother helps me address questions in my study”; “Father/mother shows physical affection to me”. In the current study, Cronbach’s alpha coefficients reached 0.96 and 0.97 for the father- and mother-targeted versions of the questionnaire, respectively.

#### Internal locus of control scale

Levenson’s IPC scale is a commonly used tool for measuring locus of control, including three dimensions: internal, powerful others and chances (Levenson, 1973). Internal locus of control was assessed by the Internality subscale from the Levenson’s IPC Scale. The examples of items are: “When I make plans, I am almost certain to make them work”; “When I get what I want, it is usually because I worked hard for it.” The Levenson’s subscale consists of eight items that are rated on a 6-point Likert scale from −3, indicating complete disagreement, to 3, indicating complete agreement. When calculating the score, 24 is added to offset the negative score, so each subscale score is 0–48. The Levenson’s subscale Cronbach’s *α* coefficient was 0.78 in the present study.

### Procedure and analysis

Data analysis was performed using SPSS 26.0, encompassing descriptive statistics, correlational analyses, and polynomial regression modeling. Response Surface Analysis (RSA) was conducted on the polynomial regression outcomes via the SPSS RSA module. Using the bootstrap resampling method (Edwards and Cable, 2009), 5,000 samples of polynomial regression coefficients from Equation 1 were drawn to estimate critical 3D response surface characteristics and test their statistical significance. Three-dimensional response surface plots were subsequently generated using Microsoft Excel.

Before formal analysis, prerequisites for RSA were verified: paternal involvement and maternal involvement were mean-centered, and the distribution of congruent and incongruent groups was confirmed to be sufficient for subsequent analysis.

Polynomial regression analysis was employed to test a set of theoretically derived linear and nonlinear equations (Edwards, 2002; Edwards and Cable, 2009). The model included independent variables *X* and *Y*, along with their quadratic terms and interaction product. This approach addresses three core theory-driven questions in centered-variable models: (1) Independent effects associated with absolute scores of *X* and *Y*; (2) Effects attributable to congruence between *X* and *Y*; (3) Effects related to the direction and magnitude of incongruence between *X* and *Y*. The quadratic polynomial regression model was specified by:


$$Z=b0+\text{control variable}+b1X+b2Y+b3X^{2}+b4\mathrm{XY}+b5Y^{2}+e$$ (1)

In this equation, *Z* represents the dependent variable, *X* and *Y* denote the two independent variables, b0 signifies the intercept, b1–b5 correspond to unstandardized regression coefficients, and *e* indicates the error term. The analytical procedure follows three sequential steps (Edwards and Cable, 2009; Shanock et al., 2010): First, predictor variables of paternal involvement (*X*) and maternal involvement (*Y*) were centered by subtracting the scale midpoint from raw values. Second, the sample’s distributional characteristics along congruence (*X* = *Y*) and directional incongruence (*X* > *Y* vs. *X* < *Y*) dimensions were examined to validate methodological appropriateness (Shanock et al., 2010). Finally, upon confirming substantive incongruence, quadratic terms (*X*^2^, *Y*^2^) and the interaction term (*X* × *Y*) were computed for polynomial regression modeling to evaluate linear, nonlinear, and interactive relationships between predictors and outcome variables.

The second stage of analysis involved hierarchical polynomial regression. Control variables alongside linear terms *X* and *Y* were initially entered into the regression equation to examine linear effects on the dependent variable *Z*. Subsequently, the interaction term *XY* and quadratic terms *X*^2^ and *Y*^2^ were incorporated to evaluate curvilinear relationships and interaction effects, with the incremental explanatory power assessed through Δ*R*^2^ significance testing. Where a statistically significant Δ*R*^2^ (*p* < 0.05) emerged concurrently with at least one significant quadratic term (Edwards, 2002), response surface analysis (RSA) was applied to the polynomial regression output.

Proceeding to the third stage, three-dimensional response surfaces were mathematically derived from the polynomial regression coefficients following Edwards (2002). Regression coefficients b1–b5 underwent transformation into RSA parameters a1–a5 (Schönbrodt et al., 2018) to facilitate interpretation of critical surface features—including stationary points, slope/curvature profiles along lines of congruence (LOC) and incongruence (LOIC), and alignment with the first principal axis. When LOC/LOIC curvature coefficients (a2 or a4) deviated significantly from zero (*p* < 0.05), comparative analyses of predicted values (*Ẑ*) were conducted between high versus low endpoints along the respective lines to quantify effect magnitudes (Shanock et al., 2010), with Cohen’s d reported for substantive interpretation.

## Results

### Common method bias test

To address potential common method bias, Harman’s single-factor test was conducted on all main variables. The unrotated factor solution showed that the first common factor accounted for 18.72% of the total variance, which was far below the 40% critical threshold, indicating no severe common method bias in the present study.

### Descriptive statistics and correlation analysis

As presented in Table 1, statistically significant gender differences emerged in paternal involvement and internal locus of control scores (*p* < 0.05), with boys demonstrating significantly higher levels than girls. No significant grade-level differences were observed for paternal involvement, maternal involvement, or internal locus of control (*p* > 0.05). Significant positive correlations were identified between internal locus of control and both paternal involvement (*p* < 0.001) and maternal involvement (*p* < 0.001).

**Table 1:** Descriptive statistics and correlations.

Variable	*M ± S*	*t_Gender_*	*F_Grade_*	PI	MI	ILOC
PI	65.07 ± 21.13	6.06*	2.59	1.00		
MI	73.32 ± 19.59	0.66	1.13	0.58***	1.00	
ILOC	28.75 ± 8.97	24.43***	2.78	0.29***	0.35***	1.00

*p < 0.05, **p < 0.01, ***p < 0.001. PI, paternal involvement, MI, maternal involvement, ILOC, internal locus of control.

It should be noted that correlational analysis only reflects general linear bivariate associations, which cannot capture the dyadic, nonlinear, and interactive effects of paternal and maternal involvement. The positive linear correlations and RSA congruence pattern are not contradictory: the linear trend reflects average associations, while RSA unpacks nuanced joint effects at different congruence levels. Therefore, Response Surface Analysis was adopted as the core method to test the research hypotheses, which can reveal the nuanced effects of involvement congruence and incongruence more accurately.

### Response surface analysis

Prior to polynomial regression analysis, the distributional characteristics of parental involvement congruence were quantified to establish analytical relevance (Shanock et al., 2010). Following variable standardization, results indicated: 17.4% (*n* = 115) exhibited high paternal–low maternal involvement, 20.2% (*n* = 133) demonstrated low paternal–high maternal involvement, 62.4% (*n* = 412) displayed parental involvement congruence. This distribution reflects multifaceted parental involvement patterns, satisfying prerequisite conditions for polynomial regression. The hierarchical regression revealed Model 2 accounted for significant variance in internal locus of control (*R^2^* = 0.16, *F* = 25.48, *p* < 0.001). Incremental validity testing of quadratic terms (*X^2^*, *XY*, *Y^2^*) showed significant *ΔR*^2^ = 0.03 (*F* = 7.25, *p* < 0.001), confirming model suitability for response surface analysis. Response Surface Analysis yielded critical parameters: a5 = 0.082 (*p* = 0.204) → First principal axis aligned with line of congruence (LOC); a4 = 0.28 (*p* < 0.001), a3 = 0.06 (*p* = 0.555) → Line of incongruence (LOIC) exhibited inverted U-shape. Collectively, these results support the broad congruence hypothesis (Humberg et al., 2019; Schönbrodt et al., 2018).

As shown in Table 2, after controlling for gender, both paternal (*p* < 0.05) and maternal involvement (*p* < 0.05) positively predicted internal locus of control. The polynomial model demonstrated a statistically significant Δ*R*^2^ (*p* < 0.001). Though incrementally small, this effect carries substantive theoretical and practical implications (Schönbrodt et al., 2018), indicating an interdependent nonlinear relationship between parental involvement and internal locus of control.

**Table 2:** Response surface analysis parameters.

Variable	Model 1		Model 2	
	*B*	*t*	*B*	*t*
Constant	0.00	0.00	−0.14	−2.98**
X (b1)	0.13	3.01**	0.23	4.16***
Y (b2)	0.27	6.14***	0.17	3.08**
X^2^ (b3)			0.14	3.26**
XY (b4)			−0.09	−1.97**
Y^2^ (b5)			0.05	1.47
LOC parameters				
a1 (b1 + b2)			0.40	9.69***
a2 (b3 + b4 + b5)			0.10	3.44**
LOIC parameters				
a3 (b1−b2)			0.06	0.59
a4 (b3−b4 + b5)			0.28	3.30**
Principal axis alignment LOC				
a5 (b3−b5)			0.08	1.27
*R*^2^	0.14		0.16	
*F*	51.35***		7.25***	
Δ*R*^2^			0.03***	

*p < 0.05, **p < 0.01, ***p < 0.001. Coefficients are unstandardized.

**Figure 1:**
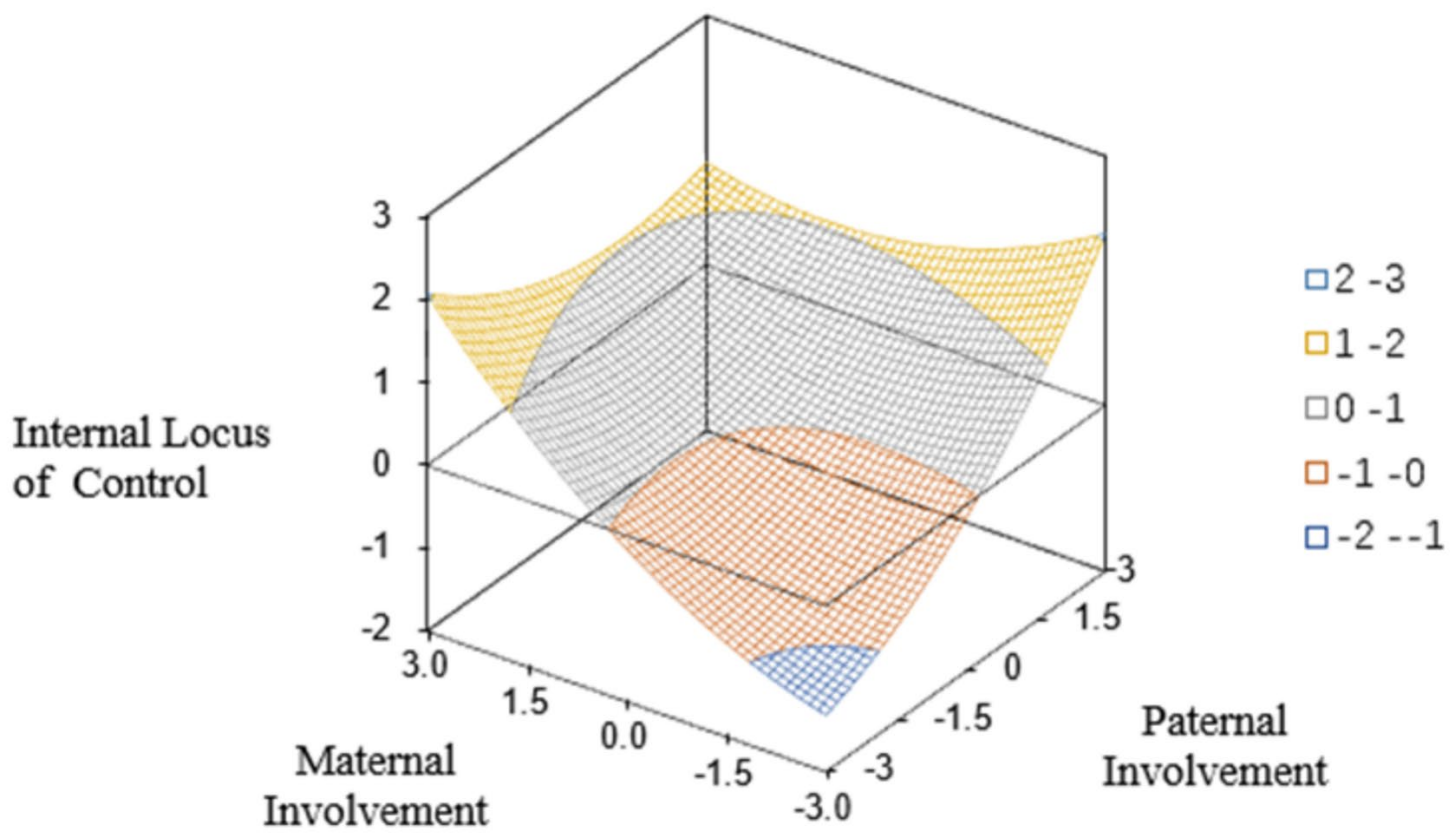
Response surface of parental involvement congruence predicting adolescents’ internal locus of control.

The nonsignificant a5 (*p* = 0.204) confirmed the second principal axis aligned perfectly with the line of congruence (LOC). Significant positive slope (a1 = 0.40, *p* < 0.001) and curvature (a2 = 0.10, *p* < 0.01) along the LOC collectively reflect two key patterns: the positive linear slope indicates a general level effect (higher overall involvement correlates with higher ILOC), while the positive curvature reveals a nonlinear diminishing marginal effect—the positive association weakens sharply at high congruence. This pattern results in a downward trend from the back corner (high-high congruence) to the front corner (low-low congruence) in the response surface (Figure 1), signifying higher internal locus of control under “low paternal–low maternal” congruence compared to “high paternal-high maternal” congruence (*Ẑ* = −0.44, 95% *CI* [−0.56, −0.32]). Notably, despite the positive level effect, the dominant nonlinear curvature and endpoint comparison support the nuanced congruence hypothesis (Humberg et al., 2019).

The significant negative curvature along the line of incongruence (a4 = 0.28, *p* < 0.01) indicates a concave downward profile traversing from left (low paternal-high maternal) to right (high paternal-low maternal). Internal locus of control decreased initially along the LOIC, reaching its nadir near the stationary point, then increased progressively. This demonstrates that adolescents’ internal locus of control varies systematically with both the direction and magnitude of parental involvement incongruence. Critically, “high paternal-low maternal” incongruence predicted significantly higher internal locus of control than “low paternal-high maternal” incongruence. Furthermore, a positive dose–response gradient emerged: larger discrepancies favoring paternal involvement (paternal > maternal) predicted incrementally higher internal locus of control.

## Discussion

The present study employed response surface analysis (RSA) to explore how congruent and incongruent patterns of paternal and maternal involvement relate to adolescents’ internal locus of control. The findings reveal nuanced dynamics that challenge conventional assumptions about parental involvement, particularly within the cultural context of Chinese families. By integrating these results with existing theories and empirical evidence, we elaborate on their implications for understanding family processes and adolescent development.

Consistent with prior research on gendered parenting in Chinese families (Liu et al., 2023; Zheng et al., 2025), boys reported significantly higher levels of paternal involvement and internal locus of control than girls. This pattern aligns with Chinese traditional gender role norms, where fathers are often socialized to show greater involvement in sons’ development, viewing them as carriers of family expectations for achievement and autonomy (Zhou, 2008; Li, 2020). When fathers provide academic guidance or decision-making support—termed “instrumental parenting” in cultural contexts—boys tend to report the belief that “effort and ability determine outcomes” (Zheng et al., 2025). Furthermore, grounded in gender role socialization theory, parental child-rearing practices are influenced by gender stereotypes, leading to differential developmental expectations for boys and girls (Hastings et al., 2005). Within traditional Chinese parenting values, parents of girls are more likely to emphasize prosocial behaviors—tending to discuss others’ emotional experiences with daughters, thereby reinforcing traits associated with heightened sensitivity to others’ needs. Conversely, parents of boys predominantly prioritize independence and exploration (Chen et al., 2014; Guo et al., 2024), demonstrating a pronounced emphasis on cultivating internal locus of control.

Both paternal and maternal involvement correlated positively with internal locus of control, aligning with earlier studies that supportive parenting fosters beliefs in personal control (Ahlin and Lobo Antunes, 2015; Yang et al., 2024). However, the RSA results reveal that this correlation is nonlinear. A striking result is that “low paternal–low maternal” parental involvement predicted higher internal locus of control than “high paternal–high maternal” involvement. This result is theoretically consistent with Rotter’s Locus of Control Theory (Rotter, 1966), which posits that internal locus of control originates from individuals’ autonomous experience and perceived control over behavioral outcomes, rather than the intensity of external parental support. In contexts of intense educational competition, “high-high” involvement often translates into coordinated pressure (e.g., fathers enforcing academic rigor while mothers managing extracurricular schedules), creating a synergistic sense of constraint. This will undoubtedly squeeze adolescents’ space for autonomous decision-making. Culturally, this dynamic is amplified by Chinese parents’ restrained emotional expression (Rothenberg et al., 2020; Rienecke, 2020; Zhou and Cui, 2025). Adolescents may interpret high involvement not as care, but as surveillance, creating a “perceptual gap” where parental intentions (support) clash with adolescent experiences (control; Leung and Shek, 2014; De Los Reyes and Ohannessian, 2016). In contrast, moderate low-low involvement (non-neglectful) conveys parental trust and provides sufficient opportunities for independent practice, which is the core condition for the development of internal locus of control (Li, 2016; Güzel et al., 2024). This result does not conflate supportive parental involvement with overcontrol (Rohner et al., 2024); instead, it reveals the perceptual outcome of excessive supportive involvement in specific cultural contexts.

Under incongruent conditions, “high paternal–low maternal” involvement outperformed “low paternal–high maternal” involvement, with larger paternal-maternal differences predicting higher internal locus of control. This underscores fathers’ unique influence in Chinese families, where traditional norms cast them as “breadwinners” rather than active caregivers (Wu et al., 2012). When fathers transcend these norms (e.g., by prioritizing childrearing over work), adolescents attribute greater significance to their involvement, interpreting it as voluntary support rather than obligatory duty (Flouri and Buchanan, 2003; Cabrera, 2020). Family systems theory (Cox and Paley, 2003) also helps explain this: high paternal involvement is associated with the family’s “expected equilibrium,” creating a dynamic where their involvement carries disproportionate psychological weight (Liu et al., 2013). Unlike maternal involvement, which is culturally taken for granted, paternal involvement, even at moderate levels, signals “extra effort,” reinforcing adolescents’ sense of being valued (Offer and Kaplan, 2021). Moreover, paternal involvement in China often focuses on autonomy-building behaviors (e.g., discussing future goals), directly strengthening internal locus of control (Zheng et al., 2025). In contrast, high maternal involvement without paternal involvement may inadvertently may be linked to greater dependency, as adolescents associate success with emotional support rather than personal agency (Leung and Shek, 2014; Pekel-Uludağlı, 2024). In addition, an increase in the gap where paternal involvement is higher than maternal involvement may enhance adolescents’ sense of responsibility through clear gender role modeling, thereby improving their internal locus of control (Cahill et al., 2024). This finding extends previous research on “parental role complementarity,” suggesting that the “directional difference” in parental involvement is more predictive than the “absolute level”.

### Limitations and future directions

Although this study has obtained some valuable findings, it still has the following limitations: First, the cross-sectional design precludes causal conclusions—we can only reveal significant associations rather than causal relationships, and longitudinal studies are needed to track the bidirectional effects between parental involvement and internal locus of control. Secondly, all data were collected through adolescent self-reports, which may lead to shared method variance and subjective perception bias; future research should integrate parent, teacher, and observational data to validate the findings. Third, the sample was recruited from urban junior high schools in Guangdong Province, which has certain cultural and regional specificity, and the findings may not be fully generalizable to rural areas, other provinces or Western cultural contexts. Finally, we did not explore mediators such as perceived autonomy or parent–child communication, which could clarify why certain involvement patterns affect internal locus of control.

Future work should also examine whether these patterns persist across developmental stages, for example, whether “low paternal–low maternal” involvement remains beneficial as adolescents enter late adolescence, and explore subgroup differences (e.g., by family socioeconomic status) to identify vulnerable populations.

## Conclusion

This study reveals that parental involvement shapes adolescents’ internal locus of control through nuanced, culturally embedded dynamics. Congruent high involvement may be linked to reduced development by restricting autonomy, while paternal involvement, especially in “high paternal–low maternal” configurations, is strongly associated with higher internal locus of control in Chinese contexts. These findings challenge simplistic notions of “good parenting” and highlight the need for culturally responsive approaches that balance support with independence. By understanding how parental roles interact, we can better foster the self-reliance that underpins healthy adolescent development.

## Data Availability

The raw data supporting the conclusions of this article will be made available by the authors, without undue reservation.

## References

[ref1] Ahlin E. M. Lobo Antunes M. J. (2015). Locus of control orientation: parents, peers, and place. J. Youth Adolesc. 44, 1803–1818. doi: 10.1007/s10964-015-0253-9, 25617000

[ref2] Ajzen I. (2002). Perceived behavioral control, self-efficacy, locus of control, and the theory of planned behavior. J. Appl. Soc. Psychol. 32, 665–683. doi: 10.1111/j.1559-1816.2002.tb00236.x

[ref3] Boonk L. Gijselaers H. J. Ritzen H. Brand-Gruwel S. (2018). A review of the relationship between parental involvement indicators and academic achievement. Educ. Res. Rev. 24, 10–30. doi: 10.1016/j.edurev.2018.02.001

[ref4] Bronfenbrenner U. (1986). Ecology of the family as a context for human development: research perspectives. Dev. Psychol. 22, 723–742. doi: 10.1037/0012-1649.22.6.723

[ref5] Cabrera N. J. (2020). Father involvement, father-child relationship, and attachment in the early years. Attach Hum. Dev. 22, 134–138. doi: 10.1080/14616734.2019.1589070, 30968742

[ref6] Cahill K. M. Updegraff K. A. McHale S. M. Umaña-Taylor A. J. Feinberg M. E. Levy R. (2024). Parenting of siblings in Latinx families during middle childhood. J. Fam. Psychol. 38, 92–103. doi: 10.1037/fam0001167, 38010800 PMC10872963

[ref7] Chen W. Y. Lu J. M. Liu L. Q. Lin W. Y. (2014). Gender differences in empathy. Adv. Psychol. Sci. 22, 1423–1434. doi: 10.3724/SP.J.1042.2014.01423

[ref8] Chukwuorji J. C. Ituma E. A. Ugwu L. E. (2018). Locus of control and academic engagement: mediating role of religious commitment. Curr. Psychol. 37, 792–802. doi: 10.1007/s12144-016-9546-8

[ref9] Cox M. J. Paley B. (2003). Understanding Families as Systems. Curr. Dir. Psychol. Sci. 12, 193–196. doi: 10.1111/1467-8721.01259

[ref10] Cui H. Bi X. Chen W. Gao T. Qing Z. Shi K. . (2023). Gratitude and academic engagement: exploring the mediating effects of internal locus of control and subjective well-being. Front. Psychol. 14:1287702. doi: 10.3389/fpsyg.2023.1287702, 38187428 PMC10768852

[ref11] de Haan A. D. Prinzie P. Jongerling J. (2018). Latent difference score modeling: a flexible approach for studying informant discrepancies. Psychol. Assess. 30, 358–369. doi: 10.1037/pas0000480, 28406670

[ref12] De Los Reyes A. Ohannessian C. M. (2016). Introduction to the special issue: discrepancies in adolescent–parent perceptions of the family and adolescent adjustment. J. Youth Adolesc. 45, 1957–1972. doi: 10.1007/s10964-016-0533-z, 27384957

[ref13] Edwards J. R. (2002). “Alternatives to difference scores: polynomial regression analysis and response surface methodology,” in Advances in Measurement and Data Analysis, eds. Drasgow F. Schmitt N. W. (San Francisco, CA: Jossey-Bass).

[ref14] Edwards J. R. Cable D. M. (2009). The value of value congruence. J. Appl. Psychol. 94, 654–677. doi: 10.1037/a0014897 19450005

[ref15] Flouri E. Buchanan A. (2003). The role of father involvement in children’s later mental health. J. Adolesc. 26, 63–78. doi: 10.1016/S0140-1971(02)00116-1, 12550822

[ref16] Frank G. Plunkett S. W. Otten M. P. (2010). Perceived parenting, self-esteem, and general self-efficacy of Iranian American adolescents. J. Child Fam. Stud. 19, 738–746. doi: 10.1007/s10826-010-9363-x

[ref17] Georgiou S. N. Ioannou M. Stavrinides P. (2017). Parenting styles and bullying at school: the mediating role of locus of control. Int. J. Sch. Educ. Psychol. 5, 226–242. doi: 10.1080/21683603.2016.1225237

[ref18] Guo J. Marsh H. W. Parker P. D. Hu X. (2024). Cross-cultural patterns of gender differences in STEM: gender stratification, gender equality and gender-equality paradoxes. Educ. Psychol. Rev. 36:37. doi: 10.1007/s10648-024-09872-3

[ref19] Güzel A. Samancı Tekin Ç. Uçan Yamaç S. (2024). Exploring the impacts of perceived locus of control on post-traumatic stress disorder among disaster survivors: a systematic review. J. Psychiatr. Ment. Health Nurs. 31, 776–787. doi: 10.1111/jpm.13030, 38340023

[ref20] Hastings P. D. Rubin K. H. DeRose L. (2005). Links among gender, inhibition, and parental socialization in the development of prosocial behavior. Merrill-Palmer Q. 51, 467–493. doi: 10.1353/mpq.2005.0023

[ref21] Humberg S. Nestler S. Back M. D. (2019). Response surface analysis in personality and social psychology: checklist and clarifications for the case of congruence hypotheses. Soc. Psychol. Personal. Sci. 10, 409–419. doi: 10.1177/1948550618757600

[ref22] Janssen L. H. C. Verkuil B. van Houtum L. A. E. M. Wever M. C. M. Elzinga B. M. (2021). Perceptions of parenting in daily life: adolescent - parent differences and associations with adolescent affect. J. Youth Adolesc. 50, 2427–2443. doi: 10.1007/s10964-021-01489-x, 34482492 PMC8580902

[ref23] Kapetanovic S. Boson K. (2022). Discrepancies in parents’ and adolescents’ reports on parent-adolescent communication and associations to adolescents’ psychological health. Curr. Psychol. 41, 4259–4270. doi: 10.1007/s12144-020-00911-0

[ref24] Lamb M. E. (2010). “The role of father involvement in child development,” in The Role of the Father in Child Development, ed. Lamb M. E. (Hoboken, NJ: Wiley).

[ref25] Leung J. T. Shek D. T. (2014). Parent–adolescent discrepancies in perceived parenting characteristics and adolescent developmental outcomes in poor Chinese families. J. Child Fam. Stud. 23, 200–213. doi: 10.1007/s10826-013-9775-5, 24482569 PMC3890555

[ref26] Levenson H. (1973). Multidimensional locus of control in psychiatric patients. J. Consult. Clin. Psychol. 41, 397–404. doi: 10.1037/h0035357 4803272

[ref27] Li X. W. (2016). The influence of fathers’ educational involvement on young children’s social adaptation. J. Clin. Psychol. 24:890–893+899. doi: 10.16128/j.cnki.1005-3611.2016.05.027

[ref28] Li X. (2020). Fathers’ involvement in Chinese societies: increasing presence, uneven progress. Child Dev. Perspect. 14, 150–156. doi: 10.1111/cdep.12375

[ref29] Liu L. S. Li Y. F. Lü Y. Li Y. W. (2013). The effect of fathers’ parenting involvement on preschool children’s social skills. Psychol. Dev. Educ. 29, 38–45. doi: 10.16187/j.cnki.issn1001-4918.2013.01.002

[ref30] Liu S. Wang X. Ying J. Shi J. Wu X. (2023). Emotional involvement matters, too: associations among parental involvement, time management and academic engagement vary with youth’s developmental phase. Br. J. Educ. Psychol. 93, 903–920. doi: 10.1111/bjep.12605, 37186306

[ref9001] Luthar S. S. Kumar N. L. Zillmer N. (2020). High-achieving schools connote risks for adolescents: Problems documented, processes implicated, and directions for interventions. American Psychologist. 75, 983. doi: 10.1037/amp0000556, 31697105

[ref31] McGue M. Elkins I. Walden B. Iacono W. G. (2005). Perceptions of the parent-adolescent relationship: a longitudinal investigation. Development 41, 971–984. doi: 10.1037/0012-1649.41.6.971, 16351340

[ref32] Naghsh Z. Hashemi Z. Kadkhodaie M. (2023). The mediating role of external locus of control in the relationship between parenting styles and emotional and academic resilience. Mental Health Res. Pract. 2, 35–45. doi: 10.22034/mhrp.2023.336490.1012

[ref33] Offer S. Kaplan D. (2021). The “new father” between ideals and practices: new masculinity ideology, gender role attitudes, and fathers’ involvement in childcare. Soc. Probl. 68, 986–1009. doi: 10.1093/socpro/spab015

[ref34] Pekel-Uludağlı N. (2024). The role of the father involvement in child development: the relationships with maternal, paternal, and child characteristics. Curr. Psychol. 43, 4085–4097. doi: 10.1007/s12144-023-04649-3

[ref35] Rienecke R. D. (2020). Emotional over involvement with adolescents: a problematic construct. Curr. Treat. Options Psychiatry 7, 162–185. doi: 10.1007/s40501-020-00205-z

[ref36] Rohner R. P. Ali S. Filus A. Senese V. P. Molaver A. Ahmed R. A. . (2024). Reliability and validity of the interpersonal rejection sensitivity scale: a multicultural study. Curr. Psychol. 43, 7986–7996. doi: 10.1007/s12144-023-04980-9

[ref37] Rothenberg W. A. Lansford J. E. Bornstein M. H. Chang L. Deater-Deckard K. Di Giunta L. . (2020). Effects of parental warmth and behavioral control on adolescent externalizing and internalizing trajectories across cultures. J. Res. Adolesc. 30, 835–855. doi: 10.1111/jora.12566, 32609411 PMC8059478

[ref38] Rotter J. B. (1966). Generalized expectancies for internal versus external control of reinforcement. Psychol. Monogr. Gen. Appl. 80, 1–28. doi: 10.1037/h0092976, 5340840

[ref39] Schönbrodt F. D. Humberg S. Nestler S. (2018). Testing similarity effects with dyadic response surface analysis. Eur. J. Personal. 32, 627–641. doi: 10.1002/per.2169

[ref40] Shanock L. R. Baran B. E. Gentry W. A. Pattison S. C. Heggestad E. D. (2010). Polynomial regression with response surface analysis: a powerful approach for examining moderation and overcoming limitations of difference scores. J. Bus. Psychol. 25, 543–554. doi: 10.1007/s10869-010-9183-4

[ref41] Steinberg L. Silk J. S. (2002). “Parenting adolescents,” in Handbook of Parenting, ed. Bornstein M. H. (Mahwah, NJ: Erlbaum).

[ref42] Timperley S. Schaughency E. Riordan J. Carroll J. Das S. Reese E. (2022). Tender shoots: effects of a preschool shared book reading preventive intervention on parent–child reading and parents’ involvement in the first year of school. Sch. Ment. Heal. 14, 238–253. doi: 10.1007/s12310-022-09505-6

[ref43] Williams K. Swift J. Williams H. Van Daal V. (2017). Raising children’s self-efficacy through parental involvement in homework. Educ. Res. 59, 316–334. doi: 10.1080/00131881.2017.1344558

[ref44] Wu X. C. Guo S. R. Liu C. Chen L. L. Guo Y. Q. (2012). The prominence of paternal functions in social changes: an analysis based on ecosystem theory. J. South Chin. Norm. Univ. 6:56–62+158.

[ref45] Wu X. C. Liu C. Zou S. Q. Hou F. (2018). Psychometric properties of the adolescence revision of parental involvement behavior questionnaire. Chin. J. Clin. Psych. 26, 647–651. doi: 10.16128/j.cnki.1005-3611.2018.04.004

[ref46] Yang F. Qian Y. Xia Z. (2024). Cognitive ability and locus of control: the effect of parental involvement on the academic performance of elementary and secondary school students. Curr. Psychol. 43, 2816–2831. doi: 10.1007/s12144-023-04572-7

[ref47] Yang Z. H. Wang J. P. (2008). A correlational study on locus of control and parental rearing patterns among junior high school students. Chin. J. Clin. Psych. 16, 401–412. doi: 10.16128/j.cnki.1005-3611.2008.04.018

[ref48] Youn J. Napolitano C. M. Han D. Lee W. Rounds J. (2023). A meta-analysis of the relations between parental support and children’s career self-efficacy in South Korea and the US. J. Vocat. Behav. 141:103839. doi: 10.1016/j.jvb.2022.103839

[ref49] Zheng T. P. Zhou X. R. Liang L. C. Yuan K. M. Bian Y. F. (2025). The relationship between parent-child perceptual differences in parental warm parenting and children’s depression and aggressive behavior. Psychol. Dev. Educ. 6, 869–880. doi: 10.16187/j.cnki.issn1001-4918.2025.06.11

[ref50] Zhou X. H. (2008). Filial piety and fraternal duty traditions and hierarchy between elders and juniors: intergenerational relationships in traditional Chinese society. Zhejiang Soc. Sci. 5, 127–128. doi: 10.14167/j.zjss.2008.05.00

[ref51] Zhou J. K. Cui L. Y. (2025). The relationship between parent–child perception discrepancy in parenting involvement and adolescents’ self-efficacy: the mediating role of parent–child trust. Chin. J. Clin. Psych. 33, 560–565. doi: 10.16128/j.cnki.1005-3611.2025.03.021

